# Equity in Choosing Wisely and beyond: the effect of health literacy on healthcare decision-making and methods to support conversations about overuse

**DOI:** 10.1136/bmjqs-2024-017411

**Published:** 2024-08-22

**Authors:** Danielle M Muscat, Erin Cvejic, Jenna Smith, Rachel Thompson, Edward Chang, Marguerite Tracy, Joshua Zadro, Robyn Linder, Kirsten McCaffery

**Affiliations:** 1 Sydney Health Literacy Lab, Sydney School of Public Health, Faculty of Medicine and Health, The University of Sydney, Sydney, New South Wales, Australia; 2 Sydney School of Public Health, Faculty of Medicine and Health, The University of Sydney, Sydney, New South Wales, Australia; 3 School of Health Sciences, Faculty of Medicine and Health, The University of Sydney, Sydney, New South Wales, Australia; 4 Institute for Musculoskeletal Health, Sydney School of Public Health, Faculty of Medicine and Health, The University of Sydney, Sydney, New South Wales, Australia; 5 NPS MedicineWise, Sydney, New South Wales, Australia

**Keywords:** Decision making, Shared decision making, Communication

## Abstract

**Objective:**

To (a) examine whether the effect of the Choosing Wisely consumer questions on question-asking and shared decision-making (SDM) outcomes differs based on individuals’ health literacy and (b) explore the relationship between health literacy, question-asking and other decision-making outcomes in the context of low value care.

**Methods:**

Preplanned analysis of randomised trial data comparing: the Choosing Wisely questions, a SDM video, both interventions or control (no intervention). Randomisation was stratified by participant health literacy (‘adequate’ vs ‘limited’), as assessed by the Newest Vital Sign.

**Main outcome measures:**

Self-efficacy to ask questions and be involved in decision-making, and intention to engage in SDM.

**Participants:**

1439 Australian adults, recruited online.

**Results:**

The effects of the Choosing Wisely questions and SDM video did not differ based on participants’ health literacy for most primary or secondary outcomes (all two-way and three-way interactions p>0.05). Compared with individuals with ‘adequate’ health literacy, those with ‘limited’ health literacy had lower knowledge of SDM rights (82.1% vs 89.0%; 95% CI: 3.9% to 9.8%, p<0.001) and less positive attitudes towards SDM (48.3% vs 58.1%; 95% CI: 4.7% to 15.0%, p=0.0002). They were also more likely to indicate they would follow low-value treatment plans without further questioning (7.46/10 vs 6.94/10; 95% CI: 0.33 to 0.72, p<0.001) and generated fewer questions to ask a healthcare provider which aligned with the Choosing Wisely questions (χ^2^ (1)=73.79, p<.001). On average, 67.7% of participants with ‘limited’ health literacy indicated that they would use video interventions again compared with 55.7% of individuals with ‘adequate’ health literacy.

**Conclusion:**

Adults with limited health literacy continue to have lower scores on decision-making outcomes in the context of low value care. Ongoing work is needed to develop and test different intervention formats that support people with lower health literacy to engage in question asking and SDM.

**WHAT IS ALREADY KNOWN ON THIS TOPIC**

- Adults with lower health literacy have shorter medical visits, ask fewer questions when seeing healthcare providers and have been shown to have difficulty understanding sets of questions designed to support shared decision-making (SDM) similar to the Choosing Wisely questions.

**WHAT THIS STUDY ADDS**

- The Choosing Wisely questions and a video preparing people to engage in SDM did not have differential effects on question-asking and decision-making outcomes based on participants’ health literacy (limited vs adequate).
- Limited health literacy was associated with increased preference to follow low value treatment plans without further questions, less positive attitudes towards SDM, lower knowledge of SDM and being less likely to generate questions aligned with Choosing Wisely topics (related to treatment risks, costs and alternatives).

**HOW THIS STUDY MIGHT AFFECT RESEARCH, PRACTICE OR POLICY**

- Research and practice should continue to develop and test different intervention formats to support people with lower health literacy—who may be more susceptible to low value care—to engage in question asking and SDM.

## Introduction

 Medical overuse and low value care—that is, care that is ineffective, harmful or confers marginal benefit at disproportionately high cost^1^—are widely acknowledged as global problems straining healthcare systems. One early initiative to combat the drivers of low value care was Choosing Wisely, a campaign to stimulate conversations between clinicians and patients about unnecessary tests, treatments and procedures.^2 3^ Originating in the USA, the ABIM Foundation’s 11-year Choosing Wisely campaign has since been adopted and adapted in multiple country contexts. However, the recent decommissioning of Choosing Wisely in countries including the USA and Australia (where Choosing Wisely was an initiative of NPS MedicineWise) has sparked debate about which aspects of the campaign were effective in achieving its goals.^4^ Such critical exploration is pertinent given that Choosing Wisely continues to thrive in other countries globally.

A central tenet of the Choosing Wisely campaign is that patients are ‘prepared to talk with their clinicians’^2^ about unnecessary tests, treatments and procedures. Given the centrality of the clinician–patient relationship to the campaign,^5^ Choosing Wisely developed five questions (here ‘the Choosing Wisely questions’) for patients to ask healthcare providers to support better conversations about unnecessary tests, medications and procedures.^6^ See table 1. Despite public availability and wide international promotion, evidence to assess the effect of the Choosing Wisely questions on patient outcomes is limited (see, for example, this single prospective, randomised controlled trial^7^), with no known evaluation among adults with varying levels of health literacy. Research has shown that adults with lower health literacy have poorer health outcomes, including increased hospitalisation, mortality and prevalence of chronic disease and risk factors for health conditions.^8^ However, they also have shorter medical visits^9^ and ask fewer questions when seeing healthcare providers.^10 11^ This includes questions regarding aspects of medical care, such as their therapeutic regimen and condition,^11^ as well as the risks and benefits of procedures or medications under consideration.^12^ Qualitative work has also identified that adults with lower health literacy may have difficulty understanding sets of questions designed to support shared decision-making (SDM),^13^ such as those within the Choosing Wisely campaign.

**Table 1:** The Choosing Wisely Australia five questions

1	Do I really need this test, treatment or procedure?
2	What are the risks?
3	Are there simpler, safer options?
4	What happens if I don't do anything?
5	What are the costs?

© NPS Medicinewise Ltd. Reproduced with permission. Visit www.choosingwisely.org.au.

Advancing health equity is considered in the quintuple aim for healthcare improvement^14^—that is, one of five aims within a framework developed to guide healthcare systems towards better outcomes and more comprehensive care.^15^ To address this fifth aim, it is not only necessary to identify disparities, but also design and implement evidence-based interventions to reduce them.^14^ In the context of health literacy, tailored interventions have been shown to support more effective communication, with the potential to reduce health inequities.^16 17^ Research has demonstrated, for example, that adults with lower health literacy can be supported to understand SDM questions when provided with further clarification^13^ or training,^18^ ranging from verbal clarification of the questions’ meaning through to more extensive training in SDM. However, the effect of different communication and SDM interventions that support question asking related to the Choosing Wisely questions is yet to be examined among adults with lower health literacy.

We conducted a randomised trial to evaluate the effect of the Choosing Wisely questions and a video to prepare patients for SDM.^19 20^ The primary outcomes of the trial were: (1) self-efficacy (confidence) to ask questions and be involved in decision-making and (2) intention to engage in SDM. Secondary outcomes included intention to follow a low-value treatment plan without further questions, SDM knowledge, positive attitude towards SDM, preparedness for SDM, acceptability of interventions and proactive intervention use. Our primary analysis^19^ identified that the Choosing Wisely questions and SDM preparation video improved intention to engage in SDM and supported participants in identifying questions which align with the Choosing Wisely campaign (with some additional benefits of the video intervention). However, neither intervention changed participant’s self-efficacy to ask questions and be involved in decision-making, intention to engage in SDM or knowledge of SDM rights.

The aim of this analysis was to examine whether the effect of the Choosing Wisely consumer questions on question asking and SDM outcomes differs based on individuals’ health literacy. We also explored the relationship between health literacy and question-asking and SDM outcomes across the whole sample in the context of low value care.

## Methods

We performed a preplanned analysis of secondary research questions using data obtained from a randomised trial described fully in our published protocol^20^ and trial manuscript.^19^ In summary, adults living in Australia were recruited from a social research database of >600 000 people in 2019, with educational quota sampling to recruit participants with lower health literacy. Specifically, we aimed to recruit equal numbers of participants with limited and adequate health literacy by over-sampling participants who had less than a university degree level of education compared with those with a university degree or greater (using a 70:30 ratio, respectively). Health literacy was assessed using the Newest Vital Sign (NVS); a validated six-item measure of functional health literacy based on interpretation of a nutrition label.^21^ NVS scores of 0 and 1 indicate a high likelihood of limited health literacy, scores of 2 and 3 indicate the possibility of limited health literacy and scores of 4–6 indicate adequate health literacy. In line with published research,^10^ we combined the first two categories such that scores of 0–3 indicated limited health literacy and scores 4–6 indicated adequate health literacy.

Consenting participants were presented with a hypothetical low value care low back pain scenario, then were randomised, stratified by health literacy to one of four trial arms: (1) Choosing Wisely questions, (2) SDM video, (3) both interventions or (4) control (no intervention). See table 2 for intervention descriptions. Low back pain was selected as the hypothetical low value care scenario given the overuse of low value care related to this condition across all income settings.^22^

**Table 2:** Intervention descriptions

	Choosing wisely questions	Preparation video
Overview	The Choosing Wisely Australia five questions (see Table 1 above).	Purpose-designed 3 min video intended to prepare patients to ask questions and participate in SDM.
Description	The Choosing Wisely Australia five questions were presented to participants via a one-page document, which lists and elaborates on the questions and provides additional guidance on their rationale and use.	The video script (available in Muscat *et al*^20^) integrated recommendations for SDM preparation as outlined by Joseph-Williams *et al*.^23^ This included making explicit what SDM is, what to expect and why it is appropriate; explaining that there are two experts in the clinical encounter; challenging attitudes that there are right and wrong decisions; reassuring patients that participation in decision-making will not result in retribution and confirming that clinicians want patient participation; and building patients’ belief in their ability to take part.
Readability	Grade nine reading level.	The transcript was developed at a grade five reading level and incorporated techniques to reduce cognitive burden.^20^
Developer	Choosing Wisely Australia, an initiative of NPS MedicineWise.	Research team.

SDM, shared decision-making.

### Outcomes

Primary and secondary outcomes are listed in table 3, in line with the published protocol.^20^ There were no changes to trial outcomes after the trial commenced. Primary outcomes as well as outcomes related to intentions and knowledge were measured pre intervention and immediately post intervention. All other outcomes were measured post intervention only.

**Table 3:** Outcomes and measurement

Outcome	Measure
Primary	
Self-efficacy to ask questions	Single item adapted from Bandura’s self-efficacy theory.^34^ Participants rated their degree of confidence to ask questions of their healthcare provider by recording a number from 0 (cannot do at all) to 100 (highly certain can do).
Self-efficacy to be involved in healthcare decision-making	Single item adapted from Bandura’s self-efficacy theory.^34^ Participants rated their degree of confidence to be involved in decisions with their healthcare provider by recording a number from 0 (cannot do at all) to 100 (highly certain can do).
Self-efficacy to ask questions and be involved in healthcare decision-making	Composite measure based on two individual items listed above.
Secondary	
Intention to engage in shared decision-making	Validated, three-item scale (Cronbach alpha=0.8^35^) measuring participants’ (1) likelihood of engaging in shared decision-making, from very unlikely (−3) to very likely (+3), (2) odds of engaging in shared decision-making, from very weak (−3) to very strong (+3) and (3) agreement with the statement ‘I intend to engage in shared decision-making’, from total disagreement (−3) to total agreement (+3). Total scores were rescaled on a scale of 0–6 and the sum of the items divided by three to derive the total score of intention.
Intention to follow the treatment plan recommended without further questioning	A single item on a 10-point scale, adapted from previous research,^36^ assessing hypothetical intention to follow the treatment plan recommended by the healthcare provider without further questioning: ‘Which best describes your intention to follow the treatment plan recommended by the doctor without asking further questions?’ (1 = ‘definitely will not’ to 10 = ‘definitely will’).
Knowledge of patients’ healthcare rights	Four questions adapted from Halawany *et al*^37^ and applied to the Australian Charter of Healthcare Rights (second edition).^38^ Participants were asked to indicate ‘yes’, ‘no’ or ‘unsure’ to show whether they think the following are patient rights: (1) ask questions and be involved in open and honest communication; (2) make choices with your healthcare provider; (3) include the people that you want in planning and decision-making; (4) get clear information about your condition, including the possible benefits and risks of different tests and treatments. A foil question will be included to detect if participants are arbitrarily selecting ‘yes’ to all questions. Scores are dichotomised into (1) all questions correct or (2) not all questions correct.
Attitude towards shared decision-making	Three-item scale adapted from Dormandy *et al*^39^ assessing participants’ perceptions of shared decision-making as beneficial/not beneficial, worthwhile/not worthwhile and important/unimportant. Each item has seven response options, forming a scale from 3 to 21. Scores were recoded such that higher scores indicate more positive attitudes towards shared decision-making. Participants responding with the highest possible score on all three questions were classified as having positive attitudes.
Preparedness for shared decision-making	Modified, eight-item version of the Preparation for Decision Making (PrepDM) Scale.^40^ The PrepDM Scale was developed to assess a participants’ perception of how useful a decision support intervention is in preparing them to communicate with their practitioner at a consultation visit and to make a health decision. Items are scored on a likert scale 1–5, from ‘not at all’ (1) to ‘A great deal’ (5), with higher scores indicating a higher perceived level of preparation for decision-making. Items were summed and the total score divided by 8.
Healthcare questions	Participants asked to write down five questions that they would ask the doctor given the hypothetical healthcare scenario.
Acceptability (intervention arms only)	Adapted from Shepherd *et al*,^41^ participants are asked to rate if they would (1) recommend the (intervention) to others and (2) use the (intervention) again on a four-point scale from 1 (definitely not) to 4 (yes, definitely).^26^ Recommendations are dichotomised into would recommend (3 and 4) and would not recommend (1 and 2).
Indicator of proactive intervention use (intervention arms only)	Proportion of participants who clicked on a link to their intervention.

### Analysis

Quantitative data analyses were conducted using Stata/IC V.16.1 (StataCorp, College Station, Texas, USA) by a study statistician blinded to the intervention allocation of participants and their level of health literacy. A p value of 0.05 was set as the threshold for statistical significance. Primary and secondary outcome data were analysed as intention-to-treat using linear regression for continuous outcomes and logistic regression for dichotomous categorical outcomes. Dichotomous variables representing the study factors (health literacy: adequate, limited × questions: yes, no × video: yes, no) and their interactions were included in models as between-subjects fixed effects, controlling for preintervention values (where available). Main effects were examined to explore the relationship between health literacy and outcomes across the whole sample. Interactions were examined to explore whether the effect of interventions on the outcomes of this study were modified by health literacy.

We used summative content analysis to analyse the healthcare questions that participants indicated they would ask the healthcare provider given the hypothetical healthcare scenario. We coded the data to assess the frequency of questions matching or close to the Choosing Wisely Australia five questions. For each of the questions, participants were given a code of ‘1’ (ie, Choosing Wisely question was among participant responses) or ‘0’ if not. The total number of responses that mapped to the Choosing Wisely five questions per participant was quantitatively compared using negative binomial regression including the study factors, health literacy adequacy and their interactions (as described above). Acceptability and proactive intervention use are summarised descriptively.

## Results

Of 1918 consenting participants, 1654 were randomised to 1 of the 4 study arms. Of those, 1439 participants (87%) provided valid responses and were included in the final analysis. The flow of participants through the trial is displayed in online supplemental figure 1. The greatest proportion of participants (27.5%) were aged between 31 and 45 years, 48.8% identified as women and 45.6% (n=656) had limited health literacy using the NVS. As shown in table 4, there were statistically significant demographic differences between those with adequate and those with limited health literacy in terms of age, level of education, private health insurance and history of back pain; a greater proportion of participants with adequate health literacy were aged younger than 61 years, had a diploma or university degree and had private health insurance. 30% of participants had a history of back pain as compared with 42.2% of those with limited health literacy.

**Table 4:** Demographic information overall and stratified by HL (limited, adequate)

		Limited HL	Adequate HL	Overall	P value
N		656	783	1439	
Age (in years)	18–30	141 (21.5%)	167 (21.3%)	308 (21.4%)	0.028
	31–45	179 (27.3%)	217 (27.7%)	396 (27.5%)	
	46–60	126 (19.2%)	193 (24.6%)	319 (22.2%)	
	61–75	174 (26.5%)	181 (23.1%)	355 (24.7%)	
	76+	36 (5.5%)	25 (3.2%)	61 (4.2%)	
Gender	Male	357 (54.4%)	377 (48.1%)	734 (51.0%)	0.072
	Female	297 (45.3%)	405 (51.7%)	702 (48.8%)	
	Non-binary	1 (0.2%)	1 (0.1%)	2 (0.1%)	
	Prefer not to say	1 (0.2%)	0 (0.0%)	1 (0.1%)	
State/territory	New South Wales	197 (30.0%)	237 (30.3%)	434 (30.2%)	0.54
	Queensland	140 (21.3%)	149 (19.0%)	289 (20.1%)	
	Victoria	175 (26.7%)	201 (25.7%)	376 (26.1%)	
	South Australia	51 (7.8%)	73 (9.3%)	124 (8.6%)	
	Australian Capital Territory	11 (1.7%)	21 (2.7%)	32 (2.2%)	
	Northern Territory	3 (0.5%)	5 (0.6%)	8 (0.6%)	
	Western Australia	60 (9.1%)	82 (10.5%)	142 (9.9%)	
	Tasmania	19 (2.9%)	15 (1.9%)	34 (2.4%)	
English main language		602 (91.8%)	736 (94.0%)	1338 (93.0%)	0.099
Education	University degree	153 (23.3%)	253 (32.3%)	406 (28.2%)	<0.001
	Diploma or certificate	169 (25.8%)	263 (33.6%)	432 (30.0%)	
	Trade apprenticeship	71 (10.8%)	47 (6.0%)	118 (8.2%)	
	Higher school certificate or leaving certificate (or equivalent)	161 (24.5%)	155 (19.8%)	316 (22.0%)	
	School certificate or intermediate certificate (or equivalent)	72 (11.0%)	59 (7.5%)	131 (9.1%)	
	No school or other qualifications	30 (4.6%)	6 (0.8%)	36 (2.5%)	
Employment status	Employed working full time	208 (31.7%)	273 (34.9%)	481 (33.4%)	0.30
	Employed working part-time	116 (17.7%)	141 (18.0%)	257 (17.9%)	
	Not employed at the moment	58 (8.8%)	70 (8.9%)	128 (8.9%)	
	Family caring/home duties	62 (9.5%)	85 (10.9%)	147 (10.2%)	
	Retired	177 (27.0%)	181 (23.1%)	358 (24.9%)	
	Studying full time	27 (4.1%)	30 (3.8%)	57 (4.0%)	
	Prefer not to answer	8 (1.2%)	3 (0.4%)	11 (0.8%)	
Private health insurance		330 (50.3%)	465 (59.4%)	795 (55.2%)	<0.001
Confidence filling out medical forms	Extremely	300 (45.7%)	419 (53.5%)	719 (50.0%)	0.002
	Quite a bit	233 (35.5%)	258 (33.0%)	491 (34.1%)	
	Somewhat	95 (14.5%)	91 (11.6%)	186 (12.9%)	
	A little bit	17 (2.6%)	13 (1.7%)	30 (2.1%)	
	Not at all	11 (1.7%)	2 (0.3%)	13 (0.9%)	
Involvement in decision making related to their health	Self	559 (85.2%)	735 (93.9%)	1294 (89.9%)	<0.001
	Doctor	516 (78.7%)	669 (85.4%)	1185 (82.3%)	<0.001
	Partner or spouse	228 (34.8%)	286 (36.5%)	514 (35.7%)	0.49
	Adult child	22 (3.4%)	13 (1.7%)	35 (2.4%)	0.038
	Friend	17 (2.6%)	8 (1.0%)	25 (1.7%)	0.023
	Brother(s) or sister(s)	10 (1.5%)	13 (1.7%)	23 (1.6%)	0.84
	Another relative	11 (1.7%)	7 (0.9%)	18 (1.3%)	0.18
	Professional carer	9 (1.4%)	1 (0.1%)	10 (0.7%)	0.005
	Court-appointed guardian	2 (0.3%)	1 (0.1%)	3 (0.2%)	0.46
	Parent(s)	55 (8.4%)	90 (11.5%)	145 (10.1%)	0.051
Back pain history		277 (42.2%)	235 (30.0%)	512 (35.6%)	<0.001
Back pain knowledge	Not much at all	127 (19.4%)	168 (21.5%)	295 (20.5%)	0.100
	A little	403 (61.4%)	497 (63.5%)	900 (62.5%)	
	A lot	126 (19.2%)	118 (15.1%)	244 (17.0%)	
Randomised group	Both	165 (25.2%)	186 (23.8%)	351 (24.4%)	0.92
	SDM video	162 (24.7%)	194 (24.8%)	356 (24.7%)	
	Choosing Wisely questions	155 (23.6%)	194 (24.8%)	349 (24.3%)	
	Control	174 (26.5%)	209 (26.7%)	383 (26.6%)	

HL, health literacy.

There was no statistical evidence that the effects of the Choosing Wisely questions or video intervention were modified by health literacy for most primary or secondary outcomes (all two-way and three-way interactions p>0.05; see table 5). However, for participants who received the video or both interventions, those with limited health literacy were more likely to indicate that they would use the interventions again compared with individuals with adequate health literacy (video: 62.0% vs 47.4%; χ^2^(1)=14.94, p<0.001; both: 73.3% vs 64%; χ^2^(1)=3.52, p=0.061). The acceptability of interventions and proactive intervention use, stratified by study arm, are presented in online supplemental table 1.

**Table 5:** Descriptive statistics for outcome measures (displayed as estimated marginal means (unless otherwise indicated) with 95% CIs) immediately post intervention, stratified by health literacy and study arm

Outcome measure	Study arm				Interaction p values		
	Preparation video	Choosing Wisely questions	Both interventions	Control	Video × health literacy	Questions × health literacy	Video × questions × health literacy
**Limited health literacy**	**(n=162)**	**(n=155)**	**(n=165)**	**(n=174)**			
Primary outcomes							
Self-efficacy to ask questions and be involved in health decision-making (0–100)	85.0 (83.7, 86.4)	85.8 (84.4, 87.2)	84.0 (82.6, 85.3)	84.7 (83.4, 86.0)	0.074	0.70	0.051
Intention to engage in SDM (0–6)	5.0 (4.9, 5.1)	4.9 (4.8, 5.0)	5.2 (5.0, 5.3)	4.9 (4.7, 5.0)	0.85	0.61	0.20
Secondary outcomes							
Intention to follow treatment plan without further questioning (0–10)	7.3 (6.7, 7.5)	7.6 (7.3, 7.9)	7.5 (7.2, 7.7)	7.5 (7.3, 7.8)	0.48	0.23	0.72
Accurate knowledge of patient’s shared decision-making rights (proportion)	0.83 (0.79, 0.87)	0.84 (0.80, 0.88)	0.79 (0.74, 0.84)	0.83 (0.79, 0.87)	0.13	0.46	0.42
Positive attitude towards SDM (proportion)	0.50 (0.42, 0.58)	0.51 (0.43, 0.58)	0.50 (0.42, 0.57)	0.44 (0.36, 0.51)	0.31	0.93	0.99
Preparedness for SDM (1–5)	3.9 (3.8, 4.1)	4.0 (3.8, 4.1)	4.0 (3.8, 4.1)	(n/a)			0.35*
**Adequate health literacy**	**(n=194)**	**(n=194)**	**(n=186)**	**(n=209)**			
Primary outcomes							
Self-efficacy to ask questions and be involved in health decision-making (0–100)	84.5 (83.2, 85.7)	84.0 (82.7, 85.2)	85.6 (84.4, 86.9)	84.3 (83.1, 85.5)			
Intention to engage in SDM (0–6)	5.1 (5.0, 5.2)	5.0 (4.9, 5.1)	5.2 (5.1, 5.3)	4.8 (4.7, 4.9)			
Secondary outcomes							
Intention to follow treatment plan without further questioning (0–10)	6.8 (6.5, 7.1)	7.1 (6.8, 7.3)	6.7 (6.4, 7.0)	7.2 (6.9, 7.4)			
Accurate knowledge of patient’s shared decision-making rights (proportion)	0.87 (0.83, 0.92)	0.91 (0.86, 0.94)	0.91 (0.87, 0.95)	0.87 (0.83, 0.92)			
Positive attitude towards SDM (proportion)	0.57 (0.50, 0.64)	0.63 (0.56, 0.70)	0.62 (0.55, 0.70)	0.51 (0.44, 0.57)			
Preparedness for SDM (1–5)	3.8 (3.7, 4.0)	3.9 (3.8, 4.0)	4.0 (3.9, 4.2)	(n/a)			

* P value represents the two-way interaction of health literacy×intervention (three levels coded as video only, questions only or both).

SDM, shared decision-making.

Compared with individuals with adequate health literacy (averaged across trial arms) those with limited health literacy were less likely to have (1) complete knowledge of patient’s SDM rights (82.1% vs 89.0%; 95% CI: 3.9% to 9.8%, χ^2^(1)=21.14, p<0.001) or (2) positive attitudes towards SDM (48.3% vs 58.1%; 95% CI: 4.7% to 15.0%; χ^2^(1)=14.08, p=0.0002). They were more likely to indicate they would follow low-value treatment plans without further questioning (7.46 vs 6.94; 95% CI:0.33 to 0.72; F(1,1430)=28.88, p<0.001) and generated fewer questions to ask a healthcare provider which aligned with the Choosing Wisely questions (χ^2^(1)=73.79, p<0.001). However, there was no statistical evidence of a main effect of health literacy for either primary outcome. See table 6.

**Table 6:** Descriptive statistics for outcome measures (displayed as estimated marginal means (unless otherwise indicated) with 95% CIs) immediately post intervention, stratified by health literacy

Outcome measure	Inadequate HL	Adequate HL	Difference	P value
Primary outcomes				
Self-efficacy to ask questions and be involved in healthcare decision-making (0–100)	84.87 (84.19, 85.55)	84.59 (83.96, 85.20)	−0.29 (−1.22, 0.63)	0.54
Intention to engage in shared decision-making (0–6)	4.96 (4.91, 5.02)	5.01 (4.96, 5.06)	0.05 (−0.03, 0.12)	0.19
Secondary outcomes				
Intention to follow treatment plan without further questioning (0–10)	7.46 (7.32, 7.60)	6.94 (6.80, 7.07)	−0.53 (−0.72 to −0.33)	<0.001
Accurate knowledge of patient’s shared decision-making rights (proportion)	0.821 (0.800, 0.843)	0.890 (0.869, 0.910)	0.068 (0.039, 0.098)	<0.001
Positive attitude towards shared decision-making (proportion)	0.483 (0.445, 0.521)	0.581 (0.547, 0.616)	0.098 (0.047, 0.150)	<0.001
Questions aligned with Choosing Wisely (count)	0.74 (0.66, 0.81)	1.29 (1.20, 1.39)	0.56 (0.44, 0.68)	<0.001

Values are collapsed across intervention arms.

HL, health literacy.

## Discussion

Findings from this analysis of randomised trial data suggest that the Choosing Wisely questions and a video preparing people to engage in SDM did not have differential effects on question-asking and decision-making outcomes based on participants’ health literacy. However, those with limited health literacy were more likely to re-engage with SDM video interventions used in this study. Across the entire sample we found that lower health literacy was associated with increased preference to follow low-value treatment plans without further questions, less positive attitudes towards SDM, lower knowledge of SDM and being less likely to ask questions related to treatment risks, costs and alternatives. There was no difference in self-efficacy to ask questions and be involved in healthcare decision-making or intention to engage in SDM between those with limited and adequate health literacy, as assessed using the NVS. However, scores on these variables were generally high overall.

Previous reviews have shown that interventions tailored to adults with lower health literacy can support more effective communication and potentially reduce health inequities.^16 17^ However, we did not find any differences in the effect of the Choosing Wisely questions or a purpose-designed SDM video on the outcomes of this study based on health literacy. The lack of difference between interventions (eg, Choosing Wisely questions vs SDM video) may be driven by the relatively short and simple nature of both interventions, both of which had high levels of acceptability. We chose to present the Choosing Wisely questions to participants via an existing Choosing Wisely Australia A4 leaflet which, in addition to listing the questions, included additional explanatory text and guidance on their rationale and use. Although the full document was written at a grade 9 reading level, the questions alone are written at grade 6.7. This may account for the lack of difference when compared with the SDM video, itself developed using best-practice strategies to reduce the grade reading level and cognitive burden.^20^ Nonetheless, the finding that there was no added value of combining both interventions for those with lower health literacy is inconsistent with underpinning theories which highlight the importance of ‘preparing’ patients for decision-making prior to ‘enabling’ SDM through the provision of a decision support tool (eg, the Choosing Wisely questions).^23^ The combined effect of interventions may have been increased if delivered by the participants’ own health provider.^23^ However, given that a 2018 Cochrane review of 87 studies (45 641 patients and 3113 healthcare professionals) concluded that ‘it is uncertain whether any interventions for increasing the use of SDM by healthcare professionals are effective because the certainty of the evidence is low or very low’^24^ additional, high quality research is needed in this area.

Although intervention effects did not differ by health literacy in the current study, to our knowledge, this is the first study to show differences in healthcare question asking and attitudes towards SDM in the context of low value care. It also builds on qualitative evidence that has shown adults with lower health literacy may not always be aware of their rights to participate in decision-making about their health.^13^ Our finding that lower health literacy was associated with increased preference to follow low-value treatment plans without further questions aligns with other Australian research which identified stronger preferences for more healthcare among men eligible for prostate-specific antigen screening who had inadequate health literacy (as compared with those with adequate health literacy).^25^ However, this finding should be interpreted in light of the significant differences in back pain history by health literacy in our study, with significantly more people with lower health literacy reporting a history of back pain. Personal experience with back pain (or lack of) may have influenced participants’ outcomes in relation to SDM.

There are several implications of this work. Foremost, policymakers and clinicians need to be alert to the fact that people with lower health literacy need more support in question asking and SDM. Our results also suggest that further work is needed to develop tailored and targeted interventions to ensure that individuals with lower health literacy are supported to be involved in healthcare decisions related to low-value care. This could be, for example, by partnering with consumers and patients cocreate messaging and testing alternative communication strategies related to the reduction of low-value care. For example, Choosing Wisely campaigns in many countries have tried to deliver the key public facing message that ‘more is not always better’ by drawing on comical non-health examples (eg, posters in clinical waiting areas of a hot dog with too much mustard; cactus with too much water) (see Born *et al*^26^ for examples). The methodology of the current study could be adopted to evaluate the effectiveness of these campaigns for adults with different levels of health literacy.

Our finding that participants with lower health literacy were more likely to indicate that they would use video interventions again suggests that there needs to be opportunities for patients (particularly those with lower health literacy) to re-engage with interventions, for example, by making them available for patients to access after their visit, or online interventions easy to download or reaccess. However, recent Australian studies with socially disadvantaged populations have shown low uptake of SMS weblinks by patients, attributing this to high mobile phone turnover and increasing awareness of online scams.^27^ The authors therefore argue that the delivery of SDM interventions in clinical waiting areas remains important.^27^ Finally, practice and policy changes in response to the COVID-19 pandemic have accelerated the adoption of telemedicine and increased the prevalence of remote visits. Given that perceptions of the impact of telemedicine on question asking and SDM are mixed both across (see, eg, Hartasanchez *et al*^28^) and within studies (see, eg, Berry *et al*^29^), more research is needed. This is particularly necessary for those with lower health literacy who are likely to be from ethnic minority or older demographic groups who experience barriers to using digital technologies when accessing care.^30^ A focus on building patient’s digital literacy skills^31^ together with the application of clear communication best practices on the part of clinicians (see Coleman^32^ for example)—which have been advocated for more broadly in relation to the expansion of telehealth—may also be important for expanding SDM in the digital space.

Strengths of this study include the randomised, stratified design and targeted recruitment of people with lower health literacy. We were able to achieve a large sample size, including a high proportion of participants with lower health literacy, through online recruitment and quota sampling. This is a particular strength given that past research has shown health literacy to be independently associated with lower research interest and consent.^33^ We also used a validated, performance-based measure of health literacy. However, findings and generalisability are limited by the hypothetical scenario, self-reported outcome measures and the controlled conditions, which meant that interventions were delivered in a way that diverges from how they would be delivered in the real world. High scores on variables such as intention to engage in SDM may not translate into behaviour in clinical practice. Finally, it is unlikely that a single vignette related to low back pain would accurately convey challenges of SDM in the context of low-value care, and so further research is needed to test intervention effects in clinical populations and settings.

## Conclusion

This study has shown that people with lower health literacy were less likely to ask questions related to treatment risks, costs and alternatives and more likely to accept low-value testing and treatment options without further questions. They also reported less positive attitudes towards SDM and less knowledge of rights related to health decision-making. Given non-significant differences in the effect of the Choosing Wisely questions and a purpose-designed SDM video on the primary outcomes of this study both overall and for people with limited health literacy, further research is needed to explore different formats to support people with low health literacy to engage in question asking and SDM in the context of low-value care and more broadly.

## Supplementary material

10.1136/bmjqs-2024-017411 online supplemental file 1

## Data Availability

Data are available on reasonable request.
