# Supplementary material for: Equity in Choosing Wisely and beyond: the effect of health literacy on healthcare decision-making and methods to support conversations about overuse
Source: BMJ Qual Saf. 2024 Aug 22;34(4):e017411. doi: 10.1136/bmjqs-2024-017411 (PMC12013561; doi:10.1136/bmjqs-2024-017411)
Supplement: online supplemental file 1 [file bmjqs-34-4-s001.pdf]

Supplementary Figure 1

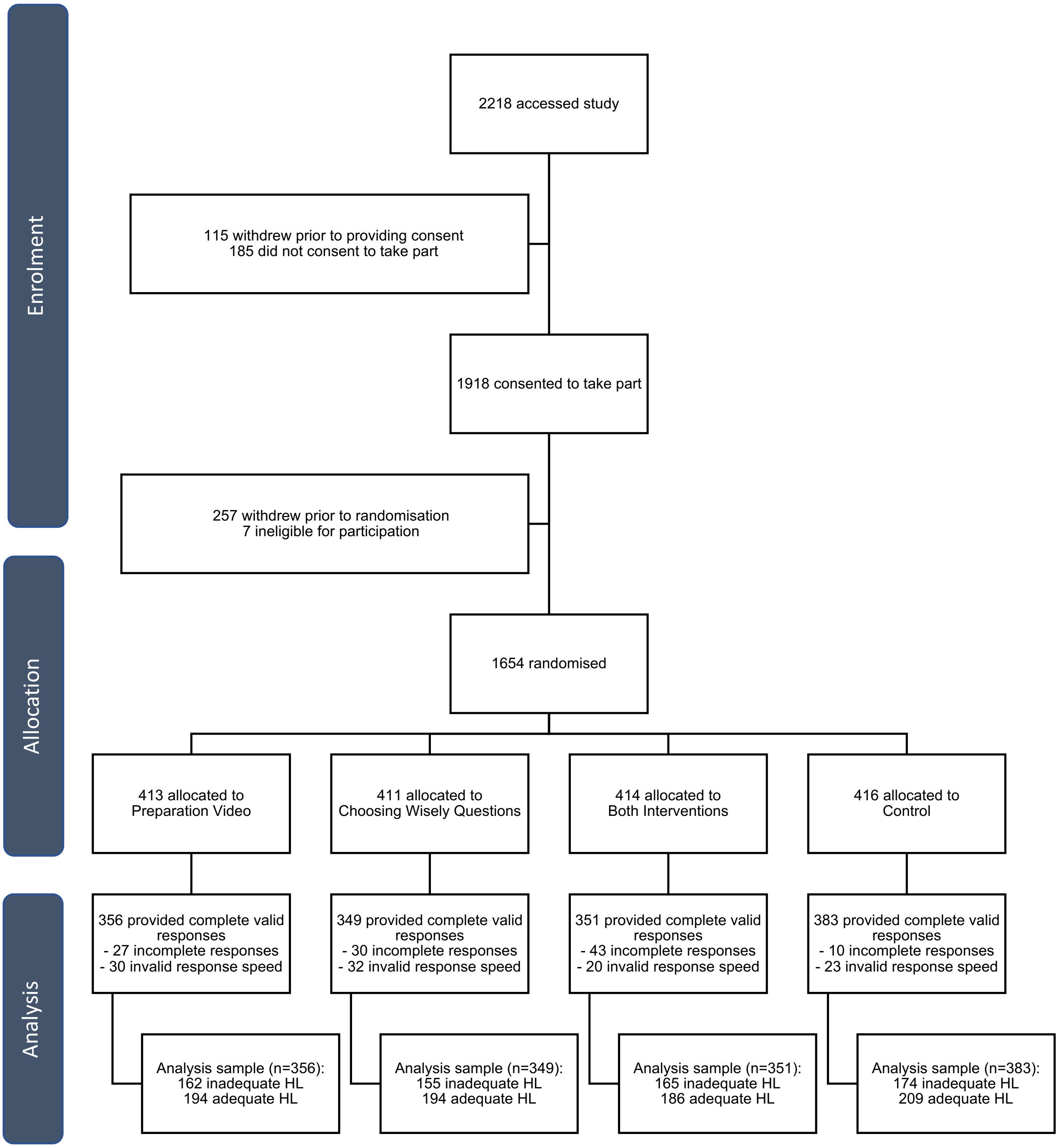

Supplementary Figure 1. Flow of participants through trial. HL = health literacy

Supplementary Table 1. Acceptability of interventions and proactive intervention use, stratified by study arm (1-3 only) and health literacy.

	Study arm				
	**Video (N=356)**	**Questions (N=349)**	**Both interventions (N=351)**		
	n (%)	n (%)	*Video* n (%)	*Questions* n (%)	*Both* n (%)
**Limited health literacy**					
Would recommend	135 (83.3)	134 (86.4)	134 (81.2)	138 (83.6)	134 (81.2)
Would use again	96 (59.3)	136 (87.7)	107 (64.8)	129 (78.2)	121 (73.3)
Proactively accessed the intervention –	7 (4.3)	7 (4.5)	4 (2.4)	4 (2.4)	2 (1.2)
**Adequate health literacy**					
Would recommend	150 (77.3)	170 (87.6)	150 (80.6)	153 (82.3)	156 (83.9)
Would use again	81 (41.7)	168 (86.6)	99 (53.2)	142 (76.3)	119 (64.0)
Proactively accessed the intervention –	5 (2.6)	23 (11.9)	8(4.3)	11 (5.9)	4 (2.2)
